# Pairwise likelihood estimation and limited‐information goodness‐of‐fit test statistics for binary factor analysis models under complex survey sampling

**DOI:** 10.1111/bmsp.12358

**Published:** 2024-10-12

**Authors:** Haziq Jamil, Irini Moustaki, Chris Skinner

**Affiliations:** ^1^ Universiti Brunei Darussalam Gadong Brunei Darussalam; ^2^ London School of Economics and Political Science London UK

**Keywords:** complex sampling, composite likelihood, factor analysis, goodness‐of‐fit tests, pairwise likelihood

## Abstract

This paper discusses estimation and limited‐information goodness‐of‐fit test statistics in factor models for binary data using pairwise likelihood estimation and sampling weights. The paper extends the applicability of pairwise likelihood estimation for factor models with binary data to accommodate complex sampling designs. Additionally, it introduces two key limited‐information test statistics: the Pearson chi‐squared test and the Wald test. To enhance computational efficiency, the paper introduces modifications to both test statistics. The performance of the estimation and the proposed test statistics under simple random sampling and unequal probability sampling is evaluated using simulated data.

## 1 INTRODUCTION

Latent variable models, such as factor analysis, are widely used in social sciences to measure abilities, attitudes, and behaviour through observed categorical (binary, ordinal) or metric variables, also known as items or indicators. There are two main approaches for modelling categorical observed variables with latent variables, namely the complete information maximum likelihood (FIML) approach used in item response theory (e.g. Bartholomew et al., 2011; Skrondal & Rabe‐Hesketh, 2004) and the limited‐information approach used in structural equation modelling (SEM) (e.g. Jöreskog, 1990; Muthén, 1984). The latter uses first‐ and second‐order statistics in univariate and bivariate likelihood functions. The limited‐information approach and, in particular, pairwise maximum likelihood (PML) are adopted here (Jöreskog & Moustaki, 2001; Katsikatsou, 2013; Katsikatsou et al., 2012; Liu, 2007; Xi, 2011). PML is a special case of composite maximum likelihood (CML) estimation (Lindsay, 1988; Varin, 2008; Varin et al., 2011). CML estimators have the desired properties of being asymptotically consistent and normally distributed. Specifically, in the case of latent variable models, PML maximizes the sum of all bivariate log‐likelihoods, which contains all the information needed for all model parameters. This approach offers a clear advantage over FIML estimation since it only requires evaluating up to two‐dimensional integrals, each integrating over a different pair of underlying variables, irrespective of the number of observed or latent variables.

Pairwise likelihood ratio tests for overall goodness of fit (GOF), nested models, and model selection criteria have been proposed by Katsikatsou and Moustaki (2016). However, due to data sparseness, overall GOF test statistics pose computational and theoretical challenges in high‐dimensional settings. To address the sparseness issues, limited‐information test statistics have been proposed. These test statistics are defined as quadratic forms of first‐ and second‐order residuals and utilize information from first‐ and second‐order marginals (Bartholomew & Leung, 2002; Cagnone & Mignani, 2007; Cai et al., 2006; Maydeu‐Olivares & Joe, 2005, 2006; Reiser, 1996, 2008). Salomaa (1990) also showed that the parameters of the factor model could be inferred from the first‐ and second‐order marginal distributions of observed variables. Pairwise likelihood estimation uses this property to estimate all model parameters from the bivariate likelihoods. Furthermore, lower‐order residuals offer valuable evidence regarding model fit. Previous research on limited‐information tests mostly used a FIML estimator and simple random sampling.

In this paper, we first extend the PML to incorporate sampling weights, thereby rendering it suitable for complex and informative sampling designs. Subsequently, we introduce a limited‐information Pearson chi‐squared test statistic alongside three variants of the Wald test statistic. These test statistics are designed to apply to simple random and complex sampling designs with unequal selection probabilities.

In complex sampling designs, the pairwise likelihood components are appropriately weighted using the provided sampling weights (Skinner, 1989). Skinner (1989) and Muthén and Satorra (1995), in the context of structural equation modelling, made a distinction between an aggregated approach, where the definitions of the model parameters take no account of complex population features such as stratification or clustering used in sampling and a disaggregated approach in which the complex population features are taken into account in the specification of the model of interest, for example, via fixed or random effect terms. Here, we focus on the aggregated approach. Skinner (2019) provides an overview of categorical data analysis for complex surveys.

The paper is organized as follows. Section 2 presents the model framework. Section 3 gives the pairwise likelihood estimation for latent variable models for categorical responses under simple random and complex survey sampling. Section 4 proposes limited‐information GOF tests for complex sampling designs. Section 5 presents the results of the simulation studies, and Section 6 concludes the paper.

## 2 FACTOR MODELS FOR BINARY DATA

The basic idea of latent variable analysis is as follows. For a given set of response variables $y_{1},\text{…},y_{p}$, one wants to find a set of latent factors $\eta_{1},\text{…},\eta_{q}$, fewer in number than the observed variables that contain essentially the same information. The latent factors are supposed to account for the dependencies of the response variables in the sense that if the factors are held fixed, the observed variables would be independent. If both the response variables and the latent factors are normally distributed with zero means and unit variances, this leads to the model (Jöreskog, 1979)

(1) $$\begin{array}{l} E(y_{i}\;|\;\eta_{1},\text{…},\eta_{q})=\lambda_{i1}\eta_{1}+\text{⋯}+\lambda_{iq}\eta_{q}, \end{array}$$

(2) $$\begin{array}{l} E(y_{i}y_{j}\;|\;\eta_{1},\text{…},\eta_{q})=0,\;i\neq j. \end{array}$$

If the factors are independent, it follows that the correlation between $y_{i}$ and $y_{j}$ is $\sum_{l=1}^{q}\lambda_{il}\lambda_{jl}$.

Equation (1) is a suitable representation of the factor analysis model if the response variables $y_{i}$ are continuous variables measured on an interval or ratio scale. However, it cannot be used if the response variables $y_{i}$ are categorical. In those cases, one must instead specify the probability of each response pattern as a function of $\eta_{1},\text{…},\eta_{q}$:

(3) $$\pi =\mathrm{Pr}(y_{1}=c_{1},\text{…},y_{p}=c_{p}|\eta_{1},\text{…},\eta_{q})=f(\eta_{1},\text{…},\eta_{q}),$$

where $c_{1},\text{…},c_{p}$ represent the different response categories of $y_{1},\text{…},y_{p}$ respectively. In this paper, we consider the case of binary response variables ($c_{i}\in \{0,1\}$) and continuous latent variables. The methodology presented can easily be extended to ordinal variables.

Let $y={\left(y_{1},\text{…},y_{p}\right)}^{\prime }$ denote the vector of p binary observed variables. Thus, there are $R=2^{p}$ possible response patterns of the form $y_{r}={\left(c_{1},c_{2},\text{…},c_{p}\right)}^{\prime }$, $c_{i}\in \{0,1\}$. For a random sample $\mathrm{34𝒮}={\left\{y^{\left(h\right)}\right\}}_{h=1}^{n}$ of size n, where $y^{\left(h\right)}$ denotes the value of y for unit h, the log‐likelihood is

(4) $$\log L(\theta ;\mathrm{34𝒮})=\overset{R}{\underset{r=1}{\sum }}n_{r}\log\pi_{r}(\theta ),$$

where $n_{r}$ and $\pi_{r}(\theta )$ are the observed frequency and the probability under the model, respectively, for the response pattern r, for some parameter vector θ. As such, $\sum_{r=1}^{R}n_{r}=n$ and $\sum_{r=1}^{R}\pi_{r}(\theta )=1$.

In the case of complex survey designs where the sample is drawn from a finite population of size N, we maximize the weighted log‐likelihood

(5) $$\log L(\theta ;\mathrm{34𝒮})=\overset{R}{\underset{r=1}{\sum }}{\hat{n}}_{r}\ln\pi_{r}(\theta ),$$

where ${\hat{n}}_{r}$ is the sum of survey weights across sample units with response pattern r. If we denote the survey weight for unit h in 34𝒮 by $w_{h}$, we can write ${\hat{n}}_{r}=\sum_{h\in \mathrm{34𝒮}}w_{h}[y^{\left(h\right)}=y_{r}]$. Here, the notation [·] denotes the Iverson bracket: [A]=1 if proposition A is true, and [A]=0 otherwise. Skinner (1989) showed that the parameter estimates from the weighted log‐likelihood (pseudo‐likelihood) are consistent under any sampling scheme.

We adopt the underlying response variable approach (UV) to model the binary variables. Each binary variable $y_{i}$ is taken to be a manifestation of an underlying continuous and normally distributed random variable $y_{i}^{*}$. The connection between the observed $y_{i}$ and the underlying variable $y_{i}^{*}$ is as follows:

$$y_{i}=\left\{\begin{array}{cc} 1\; & y_{i}^{*}>\tau_{i}\\ 0\; & y_{i}^{*}\leq \tau_{i}, \end{array}\right.$$

where $\tau_{i}$ is a threshold associated with the underlying variable $y_{i}^{*}$. For convenience, the distribution of $y_{i}^{*}$ is assumed to be standard normal.

The factor model is of the form

(6) $$y^{⋆}=\Lambda \eta +\epsilon ,$$

where $y^{⋆}={\left(y_{1}^{*},\text{⋯}\;,y_{p}^{*}\right)}^{\prime }$ is the p‐dimensional vector of the underlying variables, Λ is the p×q matrix of loadings, and ϵ is the p‐dimensional vector of unique variables. In addition, it is assumed that $\eta ∼{\text{N}}_{q}(0,\Psi )$, where Ψ contains 1s on its main diagonal. In this way, Ψ is the correlation matrix of latent factors. Further, it is assumed that $\epsilon ∼{\text{N}}_{p}(0,\Theta_{\epsilon })$, with $\Theta_{\epsilon }$ a diagonal matrix defined by $\Theta_{\epsilon }=I-\text{diag}(\Lambda \Psi \Lambda^{\prime })$, and that $\text{Cov}(\epsilon_{i},\eta_{j})=0$ for each combination of i=1,…,p and j=1,…,q.

The parameter vector $\theta^{\prime }=\left(\lambda^{\prime },\rho^{\prime },\tau^{\prime }\right)$ contains λ and ρ, which are the vectors of the free non‐redundant parameters in matrices Λ and Ψ respectively, as well as τ, which is the vector of all free thresholds. Under the UV framework, the probability of a response pattern r is written

(7) $$\pi_{r}(\theta )=\mathrm{Pr}\left(y_{1}=c_{1},\text{…},y_{p}=c_{p};\theta \right)=\int \cdot \cdot \cdot \int \phi_{p}(y^{⋆}\;|\;0,\Sigma_{y^{⋆}})\;\text{d}y^{⋆},$$

where $\phi_{p}(\cdot \;|\;\mu ,\Sigma )$ is the p‐dimensional Gaussian probability density function with mean vector μ and variance‐covariance matrix Σ. The variance‐covariance matrix of the underlying variables is the correlation matrix $\Sigma_{y^{⋆}}=\Lambda \Psi \Lambda^{\prime }+\Theta_{\epsilon }$. The p‐dimensional integral (7) is evaluated on the domain of $y^{*}$ constrained accordingly using the thresholds $\tau_{i}$, so that the manifest variables correspond to $c=(c_{1},\text{…},c_{p})$.

## 3 PAIRWISE LIKELIHOOD ESTIMATION

To mazimize the log‐likelihood function in (4) over the parameter vector θ, we need to evaluate (7), which cannot be expressed in a closed form. As a result, limited‐information estimation methods have been developed and are now widely available in commercial software. The most popular method is the three‐stage weighted least‐squares estimation (Jöreskog, 1990, 1994; Muthén, 1984). However, we will be using a different method known as PML as proposed in Katsikatsou et al. (2012) and implemented in the R package lavaan (Rosseel, 2012). The method is briefly explained in what follows.

To define the pairwise likelihood, we assume that $\pi_{c_{i}c_{j}}^{\left(ij\right)}(\theta )$ is the probability that both $y_{i}$ and $y_{j}$ will take on values $c_{i}$ and $c_{j}$ respectively under a particular model. For a random sample of size n, the pairwise log‐likelihood is

(8) $$\underset{i<j}{\sum }\underset{c_{i}=0,1}{\sum }\underset{c_{j}=0,1}{\sum }n_{c_{i}c_{j}}^{\left(ij\right)}\log\pi_{c_{i}c_{j}}^{\left(ij\right)}(\theta ),$$

where $n_{c_{i}c_{j}}^{\left(ij\right)}$ is the observed frequency of sample units with $y_{i}=c_{i}$ and $y_{j}=c_{j}$. To account for complex sampling, we use the weighted pairwise log‐likelihood instead:

(9) $$\ell_{\text{P}}(\theta ;\mathrm{34𝒮})=\underset{i<j}{\sum }\underset{c_{i}=0,1}{\sum }\underset{c_{j}=0,1}{\sum }p_{c_{i}c_{j}}^{\left(ij\right)}\log\pi_{c_{i}c_{j}}^{\left(ij\right)}(\theta ),$$

where $p_{c_{i}c_{j}}^{\left(ij\right)}=\sum_{h\in \mathrm{34𝒮}}w_{h}[y_{i}^{\left(h\right)}=c_{i},y_{j}^{\left(h\right)}=c_{j}]/\sum_{h\in s}w_{h},$ and $y_{i}^{\left(h\right)}$ refers to the ith item for sampling unit h in 34𝒮. We have introduced survey weights $w_{h}$ and scaled them by the constant factor $1/\sum_{h\in s}w_{h}$. The pairwise likelihood only requires the calculation of up to two‐dimensional normal probabilities, regardless of the number of observed or latent variables. In this way, it is always computationally feasible.

The maximum pairwise likelihood estimator (MPLE) ${\hat{\theta }}_{\text{PL}}$ solves the estimating equations ${\mathbb{R}}^{m}∋\nabla \ell_{\text{P}}(\theta ;\mathrm{34𝒮})=0$, where the entries of this score vector are

(10) $$\frac{\partial \ell_{\text{P}}(\theta ;\mathrm{34𝒮})}{\partial \theta_{k}}=\underset{i<j}{\sum }\underset{c_{i}=0,1}{\sum }\underset{c_{j}=0,1}{\sum }\frac{p_{c_{i}c_{j}}^{\left(ij\right)}}{\pi_{c_{i}c_{j}}^{\left(ij\right)}(\theta )}\frac{\partial \pi_{c_{i}c_{j}}^{\left(ij\right)}(\theta )}{\partial \theta_{k}},\;k=1,\text{…},m.$$

The (k,l) element of the m×m Hessian matrix, $\nabla^{2}\ell_{\text{P}}(\theta ;\text{y})$, is

(11) $$\frac{\partial \ell_{\text{P}}(\theta ;\mathrm{34𝒮})}{\partial \theta_{k}\partial \theta_{l}}=\underset{i<j}{\sum }\underset{c_{i}=0,1}{\sum }\underset{c_{j}=0,1}{\sum }\frac{p_{c_{i}c_{j}}^{\left(ij\right)}}{\pi_{c_{i}c_{j}}^{\left(ij\right)}(\theta )}\left\{\frac{\partial^{2}\pi_{c_{i}c_{j}}^{\left(ij\right)}(\theta )}{\partial \theta_{k}\partial \theta_{l}}-\frac{1}{\pi_{c_{i}c_{j}}^{\left(ij\right)}(\theta )}\frac{\partial \pi_{c_{i}c_{j}}^{\left(ij\right)}(\theta )}{\partial \theta_{k}}\frac{\partial \pi_{c_{i}c_{j}}^{\left(ij\right)}(\theta )}{\partial \theta_{l}}\right\}.$$

Using Taylor expansion, we may write

(12) $${\hat{\theta }}_{\text{PL}}-\theta ={\left\{\nabla^{2}\ell_{\text{P}}(\theta ;\mathrm{34𝒮})\right\}}^{-1}\nabla \ell_{\text{P}}(\theta ;\mathrm{34𝒮})+o_{p}(n^{-1/2}).$$

It follows that

(13) $$\sqrt{n}({\hat{\theta }}_{\text{PL}}-\theta )\overset{\text{d}}{\to }{\text{N}}_{m}(0,H{\left(\theta \right)}^{-1}J(\theta )H{\left(\theta \right)}^{-1}),$$

where m is the dimension of θ, $H(\theta )=\text{E}\{-\nabla^{2}\ell_{\text{P}}(\theta ;\text{y})\}$ is the sensitivity matrix, and $J(\theta )=\mathrm{Var}\left\{\nabla \ell_{\text{P}}(\theta ;y)\right\}$ is the variability matrix. $G=H(\theta )J{\left(\theta \right)}^{-1}H(\theta )$ is known as the Godambe information (Godambe, 1960) or sandwich information matrix, and it indicates the loss of efficiency compared to full information maximum likelihood estimation. If $\ell_{\text{P}}$ is a true log‐likelihood function, then the Godambe, sensitivity, and Fisher information matrices are all identical. In practice, we can estimate the H and J matrices using (Varin et al., 2011; Zhao & Joe, 2005)

(14) $$\begin{array}{l} \hat{H}({\hat{\theta }}_{\text{PL}})=-{\left.\frac{1}{\sum_{h\in \mathrm{34𝒮}}w_{h}}\underset{h\in \mathrm{34𝒮𝒮}}{\sum }\nabla^{2}\ell_{\text{P}}(\theta ;y^{\left(h\right)})\right|}_{\theta ={\hat{\theta }}_{\text{PL}}} \end{array},$$

and

(15) $$\begin{array}{l} \hat{J}({\hat{\theta }}_{\text{PL}})={\left.\frac{1}{\sum_{h\in \mathrm{34𝒮}}w_{h}}\underset{h\in \mathrm{34𝒮𝒮}}{\sum }\nabla \ell_{\text{P}}(\theta ;y_{h})\nabla \ell_{\text{P}}{\left(\theta ;y^{\left(h\right)}\right)}^{\prime }\right|}_{\theta ={\hat{\theta }}_{\text{PL}}}. \end{array}$$

The estimator for the variability matrix J assumes independent observations. However, in cluster sampling, the observations are no longer independent. The variance estimator for the weighted pairwise estimates should be adjusted to account for the dependencies and stratification (see Asparouhov, 2005; Binder, 1983; Wolter, 2007). This gives

(16) $$\hat{J}({\hat{\theta }}_{\text{PL}})=\frac{1}{n}\underset{a}{\sum }\frac{n_{a}}{n_{a}-1}\underset{b}{\sum }(z_{ab}-\bar{z_{a}}){\left(z_{ab}-\bar{z_{a}}\right)}^{\prime },$$

where $n_{a}$ is the number of sampled clusters from stratum a, $z_{ab}=\sum_{h\in {\mathrm{34𝒮}}_{ab}}\nabla \ell_{\text{P}}(\theta ;y^{\left(h\right)})$ is the total value of the score for all individuals h in cluster b in stratum a, and $\bar{z_{a}}$ is the average of $z_{ab}$. In addition, Varin (2008) discusses alternative empirical estimators for the variability matrix in the presence of clustering in a composite likelihood framework.

## 4 GOODNESS OF FIT

The overall GOF of the model can be tested by constructing a likelihood ratio test using the full log‐likelihood given in Equation (5) for testing $H_{0}:\pi_{r}=\pi_{r}(\theta )$ against $H_{1}:\pi_{r}\neq \pi_{r}(\theta )$ for all r=1,…,R, where θ is a vector of all free parameters and r runs over all possible response patterns.

Let ${\hat{\pi }}_{r}=\pi_{r}(\hat{\theta })$, where $\hat{\theta }$ denotes the FIML estimates, and let ${\hat{p}}_{r}=n_{r}/n$ denote the sample proportion of response pattern r. The maximum log‐likelihood value under $H_{0}$ is $n\sum_{r=1}^{R}p_{r}\log{\hat{\pi }}_{r}$, while the maximum log‐likelihood value under $H_{1}$ is $n\sum_{r=1}^{R}p_{r}\log p_{r}$. The likelihood ratio (LR) test statistic is

(17) $$X_{\text{LR}}^{2}=2n\overset{R}{\underset{r=1}{\sum }}p_{r}\log(p_{r}/{\hat{\pi }}_{r}).$$

Under $H_{0}$, (17) is distributed approximately as $\chi^{2}$, with degrees of freedom equal to the number of independent response patterns minus one minus the number of elements in θ. For the q‐factor model, the degrees of freedom is $2^{p}-p(q+1)-1$. Alternatively, one can use the Pearson GOF test statistic, written

(18) $$X_{\text{GF}}^{2}=n\overset{R}{\underset{r=1}{\sum }}{\left(p_{r}-{\hat{\pi }}_{r}\right)}^{2}/{\hat{\pi }}_{r}.$$

Under $H_{0}$, both test statistics (17) and (18) have the same asymptotic distribution.

In principle, these tests are possible to use with FIML. They cannot be used with the UV approach because this does not maximize an overall likelihood function, so the ${\hat{\pi }}_{r}$ are not computed. In practice, when dealing with sparse data characterised by zero or small frequencies $n_{r}$, the approximation to the chi‐squared distribution may fail and lead to unreliable results (see e.g. Reiser & VandenBerg, 1994).

To address the sparseness issue, limited‐information test statistics focus on how well the model fits first‐ and second‐order marginals (marginal proportions) such as univariate, bivariate, and trivariate rather than the entire response pattern. In the following sections, we introduce limited‐information chi‐squared test statistics for evaluating the adequacy of a parametric model under simple random sampling and complex sampling designs and pairwise likelihood estimation.

### 4.1 Definitions

Define ${\dot{\pi }}_{i}=\mathrm{Pr}(y_{i}=1)$ as the first‐order marginal proportion of a positive response for the ith binary variable i=1,…,p, and let ${\dot{\pi }}_{1}={\left({\dot{\pi }}_{1},\text{…},{\dot{\pi }}_{p}\right)}^{\prime }$ be the p×1 vector containing all such first‐order moments. Further define with ${\dot{\pi }}_{ij}=\mathrm{Pr}(y_{i}=1,y_{j}=1)$ the second‐order marginal proportion for a pair of variables i,j=1,…,p, and collect these proportions into the p2‐vector ${\dot{\pi }}_{2}={\left({\dot{\pi }}_{ij}\right)}_{i<j}$. Finally, let $\pi_{2}=\left(\begin{array}{c} {\dot{\pi }}_{1}\\ {\dot{\pi }}_{2} \end{array}\right)$be the vector that contains both these first‐ and second‐order marginal proportions with dimension S=p+p2=p(p+1)/2.

The vectors ${\dot{\pi }}_{1}$ and ${\dot{\pi }}_{2}$ are formed by summing the joint proportions of the response patterns π that satisfy specific criteria. For example, the first‐order moment ${\dot{\pi }}_{i}$ for a specific variable i is obtained by summing the joint proportions $\pi_{r}$, where the response pattern r takes the value 1 in the ith position. Similarly, the second‐order moment ${\dot{\pi }}_{ij}$ for a pair of variables i and j is obtained by summing the values of $\pi_{r}$ for which the response patterns r take on a value of 1 at both locations i and j simultaneously. In other words, there exists a transformation matrix $T_{2}:{\mathbb{R}}^{R}\to {\mathbb{R}}^{S}$ that maps the joint proportions π to the transformed vectors $\pi_{2}$, i.e. $\pi_{2}=T_{2}\pi$. This matrix consists of 0s and 1s and has a rank of S, where S represents the dimension of the transformed space. A more detailed treatment of this transformation matrix can be found in Reiser (1996) or Maydeu‐Olivares and Joe (2005).

Let p denote the R×1 vector of sample proportions based on a sample of size n, which estimates the population proportions vector π. Assuming independent and identically distributed (iid) observations, it is known that

(19) $$\sqrt{n}(\text{p}-\pi )\overset{\text{d}}{\to }{\text{N}}_{R}(0,\Sigma ),$$

where Σ is the multinomial covariance matrix given by $\Sigma =\text{diag}(\pi )-\pi \pi^{\prime }$. In the case of a complex sampling design, the vector p becomes the weighted vector of proportions with elements $\sum_{h\in \mathrm{34𝒮}}w_{h}[y^{\left(h\right)}=y_{r}]/\sum_{h\in \mathrm{34𝒮}}w_{h}$. The vector π may now be either the vector of proportions in the finite population or a superpopulation. Under suitable conditions (e.g. Fuller, 2009 sec. 1.3.2), we still have a central limit theorem as in (19), where the covariance matrix Σ does not take a multinomial form. In either case, the central limit theorem in (19) readily transforms to the limited‐information case. Consider the S×1 vectors $\pi_{2}=T_{2}\pi$ and ${\text{p}}_{2}=T_{2}\text{p}$, whose meanings are self apparent. Then

(20) $$\sqrt{n}(p_{2}-\pi_{2})\overset{\text{d}}{\to }{\text{N}}_{S}(0,\Sigma_{2}),$$

where $\Sigma_{2}=T_{2}\Sigma T_{2}^{\prime }$. Because $T_{2}$ is of full column rank S, $\Sigma_{2}$ is also of full rank S.

### 4.2 Test statistics for simple and composite hypotheses

In the case of a simple null hypothesis $H_{0}:\pi =\pi_{0}$ and iid observations, Maydeu‐Olivares and Joe (2005) proposed the test statistic

(21) $$X^{2}=n{\left(p_{2}-\pi_{20}\right)}^{\prime }\Sigma_{20}^{-1}(p_{2}-\pi_{20}),$$

where $\pi_{20}$ and $\Sigma_{20}$ are the values of $\pi_{2}$ and $\text{diag}(\pi_{2})-\pi_{2}\pi_{2}^{\prime }$, respectively, when $\pi =\pi_{0}$. As n increases, the test statistic tends towards a $\chi^{2}$ distribution with S degrees of freedom.

To evaluate the adequacy of the parametric model (composite hypothesis), we define the null hypothesis $H_{0}:\pi =\pi (\theta )$ for some θ against an alternative hypothesis $H_{1}:\pi \neq \pi (\theta )$ for any θ. To construct the test statistics, we consider the residual vector ${\hat{e}}_{2}=p_{2}-\pi_{2}({\hat{\theta }}_{\text{PL}})$, where $p_{2}$ represent the first‐ and second‐order observed proportions, while $\pi_{2}({\hat{\theta }}_{\text{PL}})$ represent the same predicted by the parametric model when evaluated at PML estimate ${\hat{\theta }}_{\text{PL}}$.

We first derive the asymptotic distribution of ${\hat{\text{e}}}_{2}$. Noting that $\pi_{2}(\theta )=T_{2}\pi (\theta )$, a Taylor series expansion about the MPLE gives

(22) $$\pi_{2}({\hat{\theta }}_{\text{PL}})=\pi_{2}(\theta )+T_{2}\Delta ({\hat{\theta }}_{\text{PL}}-\theta )+o_{p}(|{\hat{\theta }}_{\text{PL}}-\theta |),$$

where $\Delta =\frac{\partial \pi (\theta )}{\partial \theta }$. Let $\Delta_{2}$ be defined as $\Delta_{2}=\frac{\partial \pi_{2}(\theta )}{\partial \theta }=T_{2}\Delta$. Using the earlier Taylor expansion of the score vector in (12), we have that

(23) $$\begin{array}{l} {\hat{e}}_{2}=p_{2}-\pi_{2}(\theta )-\Delta_{2}{\left\{\nabla^{2}\ell_{\text{P}}(\theta )\right\}}^{-1}\nabla \ell_{\text{P}}(\theta )+o_{p}(|{\hat{\theta }}_{\text{PL}}-\theta |). \end{array}$$

To express $\nabla \ell_{\text{P}}(\theta )$ in terms of $p_{2}-\pi_{2}(\theta )$, we use 
(24)

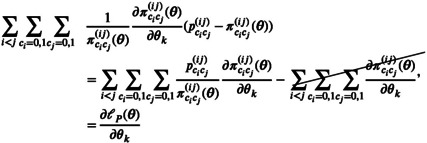

as a means of expressing the kth component of the score vector in (10). The cancellation in the last line above follows from the fact that for all k=1,…,m and any pairwise proportions with index i,j∈{1,…,p}, i<j,

(25) $$\underset{c_{i}=0,1}{\sum }\underset{c_{j}=0,1}{\sum }\pi_{c_{i}c_{j}}^{\left(ij\right)}(\theta )=1\;\;\Rightarrow \;\;\underset{c_{i}=0,1}{\sum }\underset{c_{j}=0,1}{\sum }\frac{\partial \pi_{c_{i}c_{j}}^{\left(ij\right)}(\theta )}{\partial \theta_{k}}=0.$$

Furthermore, for any pair (i,j) we require a 1×S design vector $B_{c_{i}c_{j}}^{\left(ij\right)}$ such that

(26) $$p_{c_{i}c_{j}}^{\left(ij\right)}-\pi_{c_{i}c_{j}}^{\left(ij\right)}(\theta )=B_{c_{i}c_{j}}^{\left(ij\right)}(p_{2}-\pi_{2}(\theta )),$$

since the pairwise proportions may be obtained through a linear combination of the first‐ and second‐order proportions. By substituting (26) into (24) and amalgamating the preceding terms in (24) with the design vector $B_{c_{i}c_{j}}^{\left(ij\right)}$, we find an m×S matrix B(θ) that depends on the parameters θ, which satisfies

(27) $$\nabla \ell_{\text{P}}(\theta )=B(\theta )(p_{2}-\pi_{2}(\theta )).$$

The intermediate steps from Equations (26) to (27) are given in Appendix 1. Hence, from (23), and multiplying through by $\sqrt{n}$, we get 
(28)

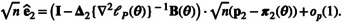

So from (20), the consistency of the Hessian, and using Slutzky's theorem, we have the limiting distribution of the lower order residuals under $H_{0}$. It is $\sqrt{n}\;{\hat{e}}_{2}\overset{\text{d}}{\to }{\text{N}}_{S}(0,\Omega_{2})$, where

(29) $$\Omega_{2}=\left(I-\Delta_{2}H{\left(\theta \right)}^{-1}B(\theta )\right)\Sigma_{2}{\left(I-\Delta_{2}H{\left(\theta \right)}^{-1}B(\theta )\right)}^{\prime }.$$

To calculate test statistics, it is necessary to use an estimator for the asymptotic covariance matrix of ${\hat{e}}_{2}$. Evaluate $\frac{\partial \pi (\theta )}{\partial \theta }$ at the PL estimate ${\hat{\theta }}_{\text{PL}}$ to obtain ${\hat{\Delta }}_{2}=T_{2}\Delta {|}_{\theta ={\hat{\theta }}_{\text{PL}}}$, and set

(30) $${\hat{\Omega }}_{2}=\left(I-{\hat{\Delta }}_{2}\hat{H}{\left({\hat{\theta }}_{\text{PL}}\right)}^{-1}\;B({\hat{\theta }}_{\text{PL}})\right){\hat{\Sigma }}_{2}{\left(I-{\hat{\Delta }}_{2}\hat{H}{\left({\hat{\theta }}_{\text{PL}}\right)}^{-1}\;B({\hat{\theta }}_{\text{PL}})\right)}^{\prime },$$

with ${\hat{\Sigma }}_{2}=T_{2}\hat{\Sigma }T_{2}^{\prime }$. The construction of the estimator $\hat{\Sigma }$ is discussed in Section 4.3. In the case of iid observations with a multinomial covariance matrix, we may set $\hat{\Sigma }=\text{diag}(\pi ({\hat{\theta }}_{\text{PL}}))-\pi ({\hat{\theta }}_{\text{PL}})\pi {\left({\hat{\theta }}_{\text{PL}}\right)}^{\prime }$.

Subsequently, the aim is to build limited‐information GOF test statistics taking the quadratic form

(31) $$X^{2}=n{\hat{e}}_{2}^{\prime }\hat{\Xi }{\hat{e}}_{2},$$

such that $\hat{\Xi }\overset{\text{P}}{\to }\Xi$ as n→∞, where Ξ is some S×S weight matrix. Generally, p‐values relating to $X^{2}$ will be compared against a reference chi‐squared distribution. This is because the quadratic form in (31) converges in distribution to $\sum_{s=1}^{S}\delta_{s}U_{s}$ as n→∞, where each $U_{s}\;\overset{\text{iid}}{∼}\;\chi_{1}^{2}$ and $\delta_{1},\text{…},\delta_{S}$ are the eigenvalues of $\Omega_{2}^{1/2}\Xi \Omega_{2}^{1/2}$ (or of $L^{\prime }\Xi L$, where L is the lower triangular matrix from the Choleski decomposition of $\Omega_{2}$). An exact asymptotic chi‐squared distribution arises if Ξ is chosen such that the eigenvalues $\delta_{s}$ are either 0 or 1. If not, a sum of scaled chi‐squared variates arises. A chi‐squared distribution can approximate this mixture of chi‐squared variates, but its degrees of freedom must be estimated. We describe in Appendix 2 the moment matching procedure to estimate the degrees of freedom of $X^{2}$ in such cases.

In what follows, we discuss Wald‐type, Pearson, and other test statistics that utilize different forms for the weight matrix Ξ.

#### 4.2.1 Wald‐type tests

A Wald test statistic can be constructed by selecting the inverse of $\Omega_{2}$ as the weight matrix Ξ. This choice ensures that the resulting test statistic follows an exact asymptotic chi‐squared distribution under the null hypothesis $H_{0}$. Due to numerical instabilities, the rank of $\Omega_{2}$ may be deficient, causing problems for calculating the inverse. In our implementation, we adopt the Moore‐Penrose inverse $\hat{\Xi }={\hat{\Omega }}_{2}^{+}$, as is done in Reiser (1996). Under the null hypothesis ($H_{0}$), this test statistic is asymptotically chi‐squared distributed with degrees of freedom equal to the rank of ${\hat{\Omega }}_{2}$, which falls between S−m and S (Maydeu‐Olivares & Joe, 2005). Here m represents all the free model parameters. One drawback is that the degrees of freedom are initially unknown and can only be estimated after estimating ${\hat{\Omega }}_{2}$ by inspecting the magnitude of its eigenvalues. As a result, the p‐value depends on the assessment of which eigenvalues are greater than zero. Additionally, inverting ${\hat{\Omega }}_{2}$ can be computationally challenging when its dimensions are large.

A different version of the Wald test is also considered, referred to as the *diagonal Wald test*, where $\hat{\Xi }=\text{diag}{\left({\hat{\Omega }}_{2}\right)}^{-1}$ is used instead of the pseudo‐inverse of $\Omega_{2}$. The motivation behind this choice is enhanced numerical stability and computational simplicity. Inverting a diagonal matrix is straightforward compared to inverting a full matrix, especially as the number of items p increases. However, the diagonal Wald test statistic converges to a sum of scaled chi‐squared values, as mentioned earlier and, in finite samples, can be approximated by an appropriate chi‐squared distribution.

The drawbacks of $\Omega_{2}$ disscused above led to Maydeu‐Olivares and Joe (2005, 2006) suggesting the use of a weight matrix Ξ such that $\Omega_{2}$ is a generalized inverse of Ξ, i.e. $\Xi =\Xi \Omega_{2}\Xi$. Let $\Delta_{2}^{⊥}$ be an S×(S−m) orthogonal complement to $\Delta_{2}$, i.e. it satisfies ${\left(\Delta_{2}^{⊥}\right)}^{\prime }\Delta_{2}=0$. Then, due to the asymptotic normality of ${\hat{e}}_{2}$, we see that as n→∞,

(32) $$\sqrt{n}{\left(\Delta_{2}^{⊥}\right)}^{\prime }{\hat{e}}_{2}\overset{\text{d}}{\to }{\text{N}}_{S-m}(0,{\left(\Delta_{2}^{⊥}\right)}^{\prime }\Omega_{2}\Delta_{2}^{⊥}).$$

Because of (29), the asymptotic covariance matrix may be written

$${\left(\Delta_{2}^{⊥}\right)}^{\prime }\Omega_{2}\Delta_{2}^{⊥}={\left(\Delta_{2}^{⊥}\right)}^{\prime }\Sigma_{2}\Delta_{2}^{⊥},$$

since all the multiplications of $\Delta_{2}$ with its orthogonal complement cancels out. By letting

$$\Xi =\Delta_{2}^{⊥}{\left({\left(\Delta_{2}^{⊥}\right)}^{\prime }\Sigma_{2}\Delta_{2}^{⊥}\right)}^{-1}{\left(\Delta_{2}^{⊥}\right)}^{\prime },$$

we can then verify $\Xi =\Xi \Omega_{2}\Xi$, that is, $\Omega_{2}$ is a generalized inverse of Ξ. Let $\hat{\Xi }$ be an appropriate estimate of Ξ, e.g. by replacing the foregoing matrices with their corresponding hat versions. The implication here is that

(33) $$X^{2}=n\;{\hat{e}}_{2}^{\prime }\hat{\Xi }{\hat{e}}_{2}=n\;{\hat{e}}_{2}^{\prime }{\hat{\Delta }}_{2}^{⊥}{\left({\left({\hat{\Delta }}_{2}^{⊥}\right)}^{\prime }{\hat{\Sigma }}_{2}{\hat{\Delta }}_{2}^{⊥}\right)}^{-1}{\left({\hat{\Delta }}_{2}^{⊥}\right)}^{\prime }{\hat{e}}_{2},$$

converges in distribution to a $\chi_{S-m}^{2}$ variate as n→∞ due to (32) and Slutsky's theorem. The degrees of freedom are as such because $\Delta_{2}^{⊥}$ is of full column rank S−m and hence Ξ is also of rank S−m, since the dimensions of a vector space and its orthogonal complement always add up to the dimension of the whole space. We refer to this test as the *variance‐covariance free (VCF) Wald test*.

#### 4.2.2 Pearson test statistic

To construct a Pearson test statistic, let ${\hat{\Xi }}^{-1}={\hat{D}}_{2}=\text{diag}(\pi_{2}({\hat{\theta }}_{\text{PL}}))$, which converges in probability to $D_{2}=\text{diag}(\pi_{2}(\theta ))$ as n→∞. Then we can see that

(34) $$X^{2}=n\;{\hat{e}}_{2}^{\prime }{\hat{D}}_{2}^{-1}{\hat{e}}_{2}=n\overset{R}{\underset{r=1}{\sum }}\frac{{\left({\dot{p}}_{r}-{\dot{\pi }}_{r}(\hat{\theta })\right)}^{2}}{{\dot{\pi }}_{r}(\hat{\theta })}+n\underset{r<s}{\sum }\frac{{\left({\dot{p}}_{rs}-{\dot{\pi }}_{rs}(\hat{\theta })\right)}^{2}}{{\dot{\pi }}_{rs}(\hat{\theta })},$$

which resembles the traditional Pearson chi‐squared test statistic. Here, the ${\dot{p}}_{r}$ and ${\dot{p}}_{rs}$ are the first‐ and second‐order sample proportion estimates to ${\dot{\pi }}_{r}$ and ${\dot{\pi }}_{rs}$ respectively. When computing the Pearson test statistic, there is no need to invert ${\hat{\Omega }}_{2}$ as in the Wald test. However, unlike the traditional Pearson test statistic, this does not follow an asymptotic chi‐squared distribution because the independence requirement is violated in the second part of the foregoing sum. Instead, it converges to a sum of scaled chi‐squared values, whose distribution may be approximated by an appropriate chi‐squared distribution. Similarly, here, we employ the moment matching adjustment described in Appendix 2 to approximate the distribution of the Pearson test statistic.

#### 4.2.3 Other test statistics

Two alternative weight matrices $\hat{\Xi }$ are proposed and selected for their ease of computation in determining the test statistic $X^{2}$. The first option is the identity matrix. This results in a chi‐squared value resembling the sum of squared residuals (RSS), which gives a pure measure of the discrepancy between the fitted model and the data. We refer to this as the RSS test.

The second option involves using multinomial weights ${\hat{\Xi }}^{-1}=\text{diag}({\hat{\pi }}_{2})-{\hat{\pi }}_{2}{\hat{\pi }}_{2}^{\prime }$. This matrix is a consistent estimator for the (inverse of) the multinomial covariance matrix $\Sigma_{2}$ for the univariate and bivariate proportions $p_{2}$. Bartholomew and Leung (2002) previously examined a variation of this test, focusing solely on the bivariate combinations and excluding the univariate ones.

In both choices mentioned here, the asymptotic distribution of the resulting test statistics is a sum of scaled chi‐squared values, so it is approximated by a chi‐squared whose degrees of freedom must be estimated using the data by employing the moment matching adjustment described in Appendix 2.

### 4.3 Estimation of covariance matrix under complex sampling

We now consider how to construct the covariance estimator of the proportion, $\hat{\Sigma }$ of Σ, in (19) for complex sampling designs. We noted earlier that under iid assumptions, we may use the multinomial expression $\hat{\Sigma }=\text{diag}(p)-pp^{\prime }$ in the simple hypothesis case and $\hat{\Sigma }=\text{diag}(\pi ({\hat{\theta }}_{\text{PL}}))-\pi ({\hat{\theta }}_{\text{PL}})\pi {\left({\hat{\theta }}_{\text{PL}}\right)}^{\prime }$ in the composite hypothesis case. We now consider the case of complex sampling, in particular, stratified multistage sampling.

From (19) we may write

$$\Sigma =\text{limvar}\{\sqrt{n}(p-\pi )\}=\text{limvar}\left\{\sqrt{n}\left(\frac{\underset{h\in \mathrm{34𝒮}}{\sum }w_{h}y^{\left(h\right)}}{\underset{h\in \mathrm{34𝒮}}{\sum }w_{h}}-\pi \right)\right\},$$

where limvar denotes the asymptotic covariance matrix, and h is the index for the units in the sample $\mathrm{34𝒮}={\left\{y^{\left(h\right)}\right\}}_{h=1}^{n}$. Using a usual linearization argument for a ratio, we may write

$$\Sigma =\text{limvar}\left\{\sqrt{n}\;\frac{\underset{h\in \mathrm{34𝒮}}{\sum }w_{h}(y^{\left(h\right)}-\pi )}{\text{E}(\underset{h\in \mathrm{34𝒮}}{\sum }w_{h})}\right\}.$$

We consider a stratified multistage sampling scheme where the strata are labelled a and the primary sampling units are labelled $b=1,\text{…},n_{a}$, where $n_{a}$ is the number of primary sampling units selected in stratum a.

Then we write

$$\frac{\underset{h\in \mathrm{34𝒮}}{\sum }w_{h}(y^{\left(h\right)}-\pi )}{\text{E}(\underset{h\in \mathrm{34𝒮}}{\sum }w_{h})}=\underset{a=1}{\sum }\underset{b=1}{\sum }{\tilde{u}}_{ab},$$

where ${\tilde{u}}_{ab}=\sum_{h\in \mathrm{34𝒮}}w_{h}(y^{\left(h\right)}-\pi )/\text{E}(\sum_{h\in \mathrm{34𝒮}}w_{h})$ and 34𝒮 is the set of sample units contained within primary sampling unit b within stratum a. So

$$\Sigma =\text{limvar}\left\{\sqrt{n}\underset{a}{\sum }\underset{b}{\sum }{\tilde{u}}_{ab}\right\}.$$

A standard estimator of $n^{-1}\Sigma$ is the between‐cluster variance estimator, which gives consistent variance estimates of linear and non‐linear statistics when the number of clusters grows large (see e.g. Asparouhov, 2005; Bieler & Williams, 1995; Binder, 1983; Skinner, 1989; Williams, 2000; Wolter, 2007). This estimator is given by

$$n^{-1}\hat{\Sigma }=\overset{M}{\underset{a=1}{\sum }}\frac{n_{a}}{n_{a}-1}\overset{n_{a}}{\underset{b=1}{\sum }}({\text{u}}_{ab}-{\bar{\text{u}}}_{a}){\left(u_{ab}-{\bar{u}}_{a}\right)}^{\prime },$$

where $n_{a}$ is the number of sampled clusters from stratum a, M is the total number of strata, $u_{ab}=\sum_{h\in s}w_{h}(y^{\left(h\right)}-\pi_{0})/\sum_{h\in s}w_{h}$ is the total for all individuals h in cluster b in stratum a, and ${\bar{\text{u}}}_{a}=n_{a}^{-1}\sum_{b=1}^{n_{a}}u_{ab}$ is the average of $u_{ab}$. Here, $\pi_{0}$ refers to the value of π under a simple null hypothesis or estimates of the model‐implied proportions π(θ) under a composite null hypothesis.

In the case of simple random sampling, we have just one stratum, the clusters are elements, and the weights are constant so that $n_{a}=n$, $u_{ab}=({\text{y}}^{\left(h\right)}-\pi_{0})/n$, ${\bar{\text{u}}}_{a}=0$, and the estimator reduces to $\frac{n}{n-1}\left(\text{diag}(\pi_{0})-\pi_{0}\pi_{0}^{\prime }\right)$.

We only require ${\hat{\Sigma }}_{2}=T_{2}\hat{\Sigma }T_{2}^{\prime }$ to compute the Wald and Pearson test statistics. We may write

$$n^{-1}{\hat{\Sigma }}_{2}=\overset{M}{\underset{a=1}{\sum }}\frac{n_{a}}{n_{a}-1}\overset{n_{a}}{\underset{b=1}{\sum }}({\text{v}}_{ab}-{\bar{\text{v}}}_{a}){\left({\text{v}}_{ab}-{\bar{\text{v}}}_{a}\right)}^{\prime },$$

where ${\text{v}}_{ab}=\sum_{h\in \mathrm{34𝒮}}w_{h}({\text{y}}_{2}^{\left(h\right)}-\pi_{20})/(\sum_{h\in \mathrm{34𝒮}}w_{h})$, ${\bar{\text{v}}}_{a}=n_{a}^{-1}\sum_{b=1}^{n_{a}}{\text{v}}_{ab}$, and $y_{2}^{\left(h\right)}=T_{2}y^{\left(h\right)}$ is the S×1 indicator vector containing values $\left[y_{i}^{\left(h\right)}=1\right]$ and $\left[y_{i}^{\left(h\right)}=y_{j}^{\left(h\right)}=1\right]$ for different values of i and j.

## 5 SIMULATION STUDY

Our simulation study comprises three parts. The first part (Part A) focuses on assessing the performance of the parameter estimates and their asymptotic standard errors in bias and mean squared error under an informative sampling scheme. These evaluations are carried out for the weighted pairwise likelihood, which accounts for unequal selection probabilities due to sampling design or informative sampling. The simulation's second and third parts (Parts B and C) study the performance of the proposed test statistics about Type I error and power for simple random sampling (SRS) and complex sampling respectively.

We made a deliberate choice to include simulation results for SRS. This was done to evaluate the performance of the proposed test statistics under limited‐information estimation methods. Research related to limited‐information GOF test statistics has primarily focused on full‐information maximum likelihood methods, as these produce the best asymptotically normal (BAN) estimators with the lowest asymptotic variance. In contrast, limited‐information estimation methods do not yield BAN estimators.

Additionally, we investigate the degree to which the null distribution of the test statistics approximate the assumed theoretical distribution as we increase the sample size and the number of observed variables.

### 5.1 Simulation Part A: Assessing parameter estimates and standard errors for the weighted pairwise likelihood

The data were generated as follows. A population of size N was created under the one‐factor model using the specified true parameter values. The size of N varied according to the size of the sample n to keep the sample‐to‐population ratio small and constant at n/N=1% to avoid the need for any finite population correction factors. Each individual indexed by h was assigned a probability of selection denoted by $\pi_{h}$, which was set at $\pi_{h}=1/(1+\exp(y_{1}^{*}))$ based on the first underlying variable $y_{1}^{*}$. Thus, a larger value of $y_{1}^{*}$ resulted in a smaller probability of selection. A similar sampling scheme was described in Asparouhov (2005). The one‐factor model was subsequently estimated using two different approaches, namely the unweighted PML given in (4) and the weighted PML (PMLW) given in (5). The sampling and estimation were replicated 1000 times and repeated for three sample sizes: n=500,1000,5000.

Table 1 shows the parameter bias for different sample sizes. When the weights from the informative sampling are considered (PMLW), the bias is minimal and close to zero. Ignoring the weights (PML) leads to a more significant bias for the threshold estimates.

**TABLE 1:** Simulation A: Bias of estimated factor loadings and thresholds for one‐factor model under informative sampling scheme, n=500,1000,5000, unweighted PML and PMLW.

	True values	n=500		n=1000		n=5000	
		PML	PMLW	PML	PMLW	PML	PMLW
*Loadings*							
$\lambda_{1}$	.80	−.026	−.002	−.030	−.006	−.017	.005
$\lambda_{2}$	.70	−.026	−.007	−.020	−.002	−.029	−.005
$\lambda_{3}$	.47	−.020	−.001	−.024	−.004	−.024	−.003
$\lambda_{4}$	.38	−.017	−.001	−.019	−.002	−.017	.003
$\lambda_{5}$	.34	−.003	.010	−.019	−.002	−.022	−.006
*Thresholds*							
$\tau_{1}$	−1.43	.309	−.004	.311	.000	.304	−.007
$\tau_{2}$	−.55	.215	−.006	.218	.002	.213	−.008
$\tau_{3}$	−.13	.145	−.006	.161	.009	.153	−.001
$\tau_{4}$	−.72	.123	.003	.117	−.001	.120	−.002
$\tau_{5}$	−1.13	.112	.009	.114	.009	.106	.002

Table 2 gives the coverage and ratio of the standard deviation of the estimated parameters across replications to the estimated asymptotic standard error. The latter was calculated using the Godambe information matrix. The coverage is .95 for most parameters regardless of the sample size under the PMLW estimation. Furthermore, the estimated asymptotic standard errors computed using the Godambe information matrix closely match the standard deviations computed across 1000 replications. Ignoring the weights during estimation impacts coverage and standard errors, leading to incorrect inferences.

**TABLE 2:** Simulation A: Coverage rate and ratio of standard deviation across replications to estimated asymptotic standard error (SD/SE) for factor loadings and thresholds for one‐factor model under an informative sampling scheme, n=500,1000,5000, PML and PMLW.

	n=500				n=1000				n=5000			
	Coverage		SD/SE		Coverage		SD/SE		Coverage		SD/SE	
	PML	PMLW	PML	PMLW	PML	PMLW	PML	PMLW	PML	PMLW	PML	PMLW
*Loadings*												
$\lambda_{1}$	.95	.96	1.01	.99	.94	.95	1.04	1.01	.89	.95	1.22	1.01
$\lambda_{2}$	.95	.95	1.03	.99	.93	.95	1.09	1.01	.85	.95	1.31	.99
$\lambda_{3}$	.95	.96	1.02	.97	.94	.95	1.06	.97	.85	.95	1.35	.99
$\lambda_{4}$	.94	.94	1.04	1.03	.94	.95	1.05	1.00	.88	.95	1.25	1.01
$\lambda_{5}$	.95	.95	.99	.98	.95	.96	1.02	1.00	.90	.95	1.19	1.01
*Thresholds*												
$\tau_{1}$	.01	.96	4.38	.98	.00	.96	6.24	.97	.00	.96	13.81	.97
$\tau_{2}$	.04	.95	3.91	1.05	.00	.94	5.47	1.01	.00	.94	12.03	1.06
$\tau_{3}$	.22	.96	2.93	1.02	.03	.94	4.03	1.02	.00	.96	8.76	.96
$\tau_{4}$	.49	.94	2.22	1.03	.20	.95	2.97	1.03	.00	.95	6.40	1.04
$\tau_{5}$	.61	.94	1.88	1.02	.42	.95	2.39	1.01	.00	.95	5.13	1.04

### 5.2 Simulation Part B: Assessing performance of limited‐information test statistics under simple random sampling

We evaluate the performance of the six test statistics outlined in Table 4 under simple random sampling (SRS) and PML. We study five different models described in Table 3 with varying numbers of items (p) and factors (q) and five sample sizes: n=500,1000,2500,5000, and 10,000. This results in 25 simulation conditions. We conducted 1000 replications for each simulation condition.

**TABLE 3:** Simulations B and C: Models used for generating the data.

Model	Label	Number of variables (p) and factors (q)
1	1F 5V	p=5 and q=1
2	1F 8V	p=8 and q=1
3	1F 15V	p=15 and q=1
4	2F 10V	p=10 and q=2, 5 indicators per factor
5	3F 15V	p=15 and q=3, 5 indicators per factor

In Model 1, the true factor loadings $\lambda^{\prime }$ are (0.8,0.7,0.47,0.38,0.34). The true threshold values $\tau^{\prime }$ are (−1.43,−0.55,−0.13,−0.72,−1.13). For Model 2, the factor loading values are identical to those in Model 1 for the first five items. Additionally, the first three factor loadings (.8, .7, .47) from Model 1 are repeated to set the values for the last three items in Model 2. The threshold values follow the same pattern. The same mechanism is used to set the true values for Model 3. Models 4 and 5 are confirmatory factor analysis models. The true factor loadings for each factor are used in Model 1. The same goes for the thresholds. For Models 4 (two factors) and 5 (three factors), the factor correlations are ρ=0.3 and $\rho^{\prime }=(0.2,0.3,0.4)$ respectively.

In each replication within each condition, we compute the GOF test statistic using Equation (31), and the weight matrices used are summarized in Table 4. The moment matching adjustment described in Appendix 2 has been applied to all tests but the Wald and VCF Wald. The degrees of freedom for the Wald test statistics are in the range (S−m,S). We found that S−m gave the best results in approximating the test statistic distribution, which we used in all our simulations. Our comparison focuses on the Wald, VCF Wald, and Pearson test statistics. The study also includes the performance of simpler versions of the tests (WaldDiag, RSS, and multinomial). However, we recommend using these simpler versions independently only after further investigations to determine their suitability in any model setting.

**TABLE 4:** Summary of test statistics evaluated in Simulations B and C.

	Name	Weight matrix (Ξ)	df
1	Wald	$\Omega_{2}^{+}$	S−m,S
2	VCF Wald	$\Xi \Omega_{2}\Xi$	S−m
3	Wald diagonal	$\text{diag}{\left(\Omega_{2}\right)}^{-1}$	Est.
4	Pearson	$\text{diag}{\left(\pi_{2}\right)}^{-1}$	Est.
5	RSS	I	Est.
6	Multinomial	${\left[\text{diag}(\pi_{2})-\pi_{2}\pi_{2}^{\prime }\right]}^{-1}$	Est.

To conduct a power analysis, we introduced an additional latent variable z∼N(0,1) independent of the latent variables η. This extra variable was included in the data‐generating model to capture together with η the associations among the $y^{*}$ variables. The loadings of variable z in the data‐generating model closely resembled the loadings of the true latent factor η. For Models 1 and 2, the extra latent variable z loads onto all items except two ($y_{2}$ and $y_{6}$). For Model 3, z loads onto all items except three ($y_{2},y_{6},y_{14}$). For Models 4 and 5, z loads onto all items. This additional latent variable in the data‐generation process results in a misspecified model, and the power of the test correctly detects the misspecification.

**FIGURE 1:**
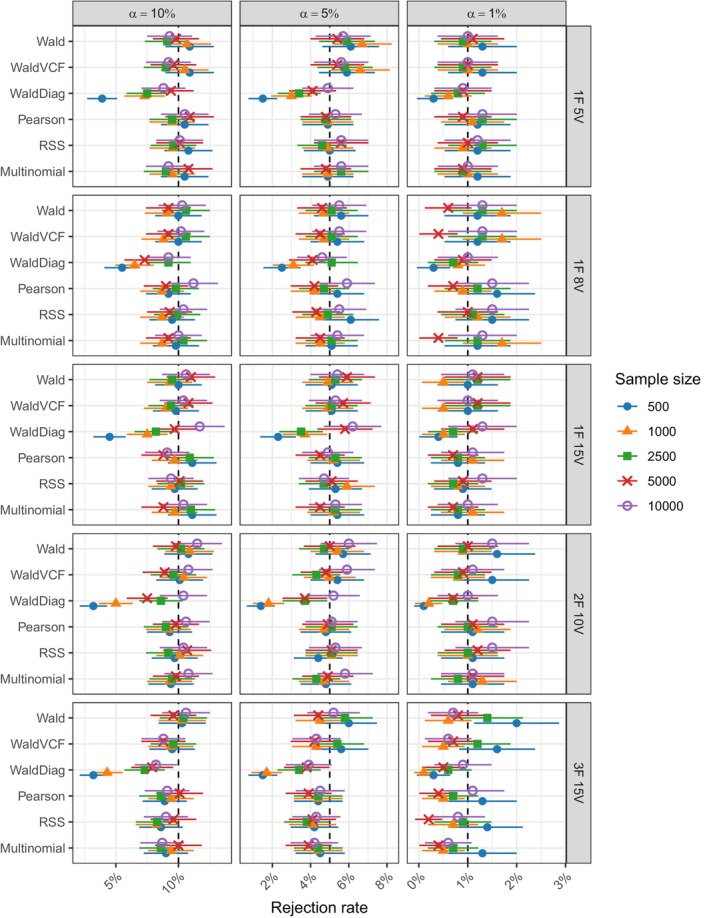
Simulation B: Type I error rates at significance levels α=(10%,5%,1%) for the six test statistics in Table 4 across the five models in Table 3 and n=500, 1000, 2500, 5000, 10,000, SRS. The reported intervals are the 95% confidence intervals for each rejection proportion.

**FIGURE 2:**
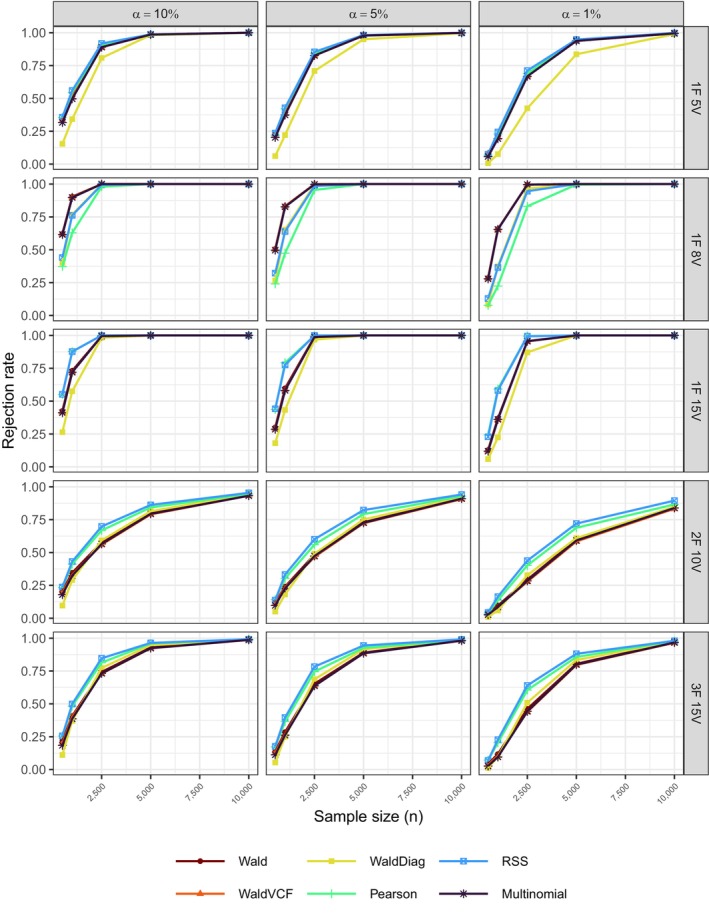
Simulation B: Power analysis at significance levels α=(10%,5%,1%) for the six test statistics in Table 4 across the five models in Table 3, and n=500, 1000, 2500, 5000, 10,000, SRS.

Figure 1 gives the Type I error rates for the six test statistics across all simulation conditions. The Wald diagonal test statistic exhibited the poorest performance. In contrast, Pearson and the Wald‐type test statistics demonstrated satisfactory performance across all three significance levels α=10%,5%, and 1%, with improvement noted as the sample size increased. The power of all tests, as shown in Figure 2, increased with sample size, although it remained at lower levels in the case of the two‐ and three‐factor models. In addition, as the complexity of the model increased, the power of the Wald‐type test statistics consistently decreased compared to the Pearson test statistic. This could be attributed to the instability in inverting the ${\hat{\Omega }}_{2}$ matrix and potential issues with its estimation arising from possible sparsity in the lower‐order margins and smaller sample sizes.

### 5.3 Simulation C: Assessing performance of limited‐information test statistics under complex sampling

We generated data for an entire population, mirroring a commonly employed sampling approach in educational surveys. The population comprised 2000 schools, acting as primary sampling units (PSUs). These schools were stratified into three types: A (400 units), B (1000 units), and C (600 units).

The schools were of different sizes so that an unequal probability sample might be conducted for the clustered sample scenario based on each school's size. The number of students allocated to each school followed a normal distribution, with a mean of 500 and a standard deviation of 125. Each school's assigned students was then rounded to the nearest whole number.

Additionally, students within each school type were randomly distributed among classes, where the average class sizes were set at 15, 25, and 20 for school Types A, B, and C respectively. The simulated population comprised approximately one million students (clustered within classrooms and classrooms clustered within schools). The population statistics, encompassing the count of schools, school types, and average class sizes, are given in Table 5.

**TABLE 5:** Simulation C: Population statistics for simulated school population.

Schools		Students					Class size			
Type	N	N	Avg.	SD	Min.	Max.	Avg.	SD	Min.	Max.
A	400	200,670	501.7	96.9	253	824	15.2	3.9	3	36
B	1000	501,326	501.3	100.2	219	839	25.7	5.0	6	48
C	600	303,861	506.4	101.9	195	829	20.4	4.4	6	39

The item responses were generated based on the five factor models outlined in Table 3. The PSUs (schools) were stratified into distinct strata based on the latent vector η, representing the abilities of each student. The observation units were ordered in descending order based on their latent variable value for the first factor $\eta_{1h}$. Subsequently, they were allocated to school Types A, B, or C. If the vector of observed variables y represents test items, this categorization implies that school Type A comprises ‘high‐ability’ students, Type B ‘medium‐ability’ students, and Type C ‘low‐ability’ students. This data‐generating scheme resulted in varying intraclass correlations (ICC) for response items across PSUs, ranging from .05 to .6. Of note, Hedges and Hedberg (2007) found that a typical value for the ICC in an educational study was about .16, adjusted for covariates.

To obtain a sample from this simulated population, we explored two multistage probability sampling designs: (1) two‐stage cluster sampling and (2) stratified cluster sampling. The following discussion details the process of selecting a sample of (approximate) size n from each design. Table 6 shows the number of clusters (PSUs) sampled using each sampling design discussed below.

1. Two‐stage cluster sampling
  Let $n_{c}=\lfloor \frac{n}{21.5}\rfloor$, where 21.5 represents the average class size across all types of schools in the population. The value of $n_{c}$ approximates the number of schools (PSUs). Schools are selected using probability proportional to size (PPS) sampling, where the size variable is the number of students in each school. Next, one class is selected using simple random sampling from each selected PSU. All students in the selected class are included in the sample. The probability of selecting a student from school b is
$$\mathrm{Pr}(\text{selection})=\frac{z_{b}}{\underset{b}{\sum }z_{b}}\times \frac{1}{M_{b}},$$
  where $z_{b}$ is the number of students in school b, $\sum_{b}z_{b}$ is the total number of students in all schools in the population, and $M_{b}$ is the number of classes in school b.
  The total sample size varies from sample to sample due to varying school and class sizes. However, on average, it is expected to be close to $n=n_{c}\times 21.5$, where 21.5 is the average number of students per class.
2. Stratified cluster sampling
  Select a fixed number of schools (PSUs) denoted by $n_{c}$ using SRS from each stratum. The quantity $n_{c}$ is determined using the formula $n_{c}=\lfloor n/(15+20+25)\rfloor$, since we want the sample size n to be close to $15n_{c}+20n_{c}+25n_{c}$, where the numbers 15, 20, and 25 represent the average class size in each stratum. One class is selected from each selected school using SRS. All the students from the selected class are included in the sample.
  The inclusion probability for a student from school, b within stratum a, is calculated to be
$$\mathrm{Pr}(\text{selection})=\frac{n_{a}}{N_{a}}\times \frac{n_{ab}}{N_{ab}}\times \frac{n_{abc}}{N_{abc}},$$
  where $n_{a}$ ($N_{a}$) is the sample (population) number of schools in stratum a, $n_{ab}$ ($N_{ab}$) is the sample (population) number of classes in school b within stratum a, and $n_{abc}$ ($N_{abc}$) is the sample (population) number of students in class c, school b within stratum a. In our experiment, $n_{a}$ was chosen to be the same in each stratum (equal allocation) denoted by $n_{c}$, and all students were selected from the selected classes, $n_{abc}=N_{abc}$.

**TABLE 6:** Number of clusters (PSUs) sampled with each sampling method.

Sampling method	n=500	n=1000	n=2500	n=5000	n = 10,000
Cluster	23	47	116	233	465
Stratified cluster	24	51	126	249	501

For the two‐stage cluster sampling design, we provide additional results in Appendix 3. Table A1 shows the coverage and the SD/SE ratio for each estimated parameter obtained from 1000 replications over the estimated asymptotic standard error for all model parameters. This is for the one‐factor model with five items, using weighted pairwise likelihood and considering clustering in estimating the variability matrix J (16) in the Godambe information matrix. The results show that when clustering is considered in estimating the Godambe information matrix, the standard deviation to standard error ratio is much closer to one than when it is not accounted for.

**FIGURE 3:**
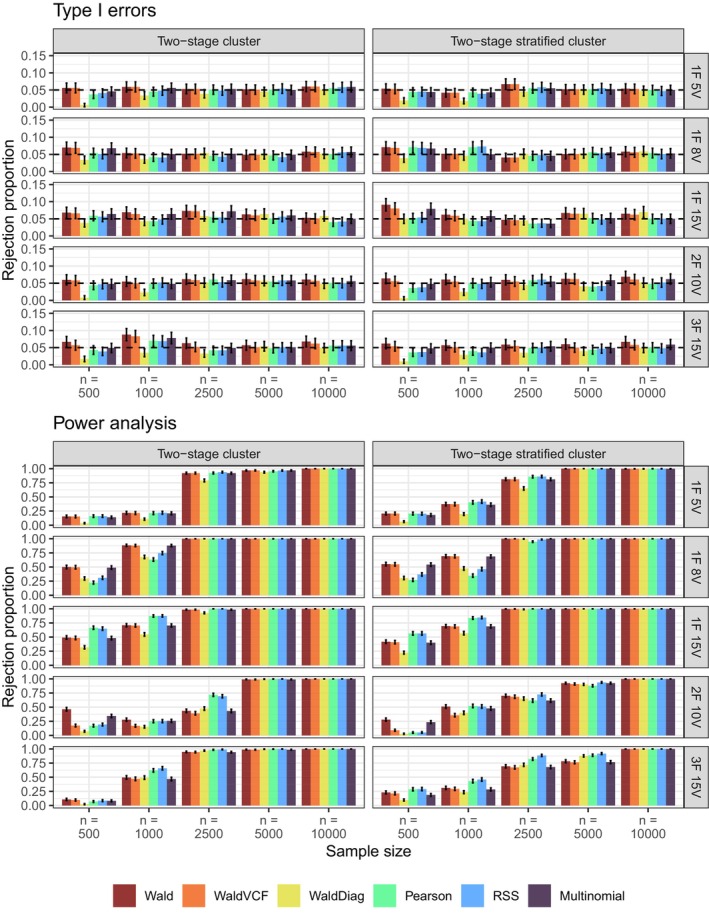
Simulation C: Type I error rates (top) and power analysis (bottom) for the two complex sampling designs at significance level α=5% for the six test statistics in Table 4 across the five models in Table 3 and n=500,1000,2500,5000,10,000. The reported intervals are the 95% confidence intervals for each rejection proportion.

Figure 3 displays the Type I error and power rates for the six test statistics under the two complex sampling designs across the five models outlined in Table 3. All test statistics performed well under both complex designs, except for the WaldDiag, which required larger sample sizes. Due to its poor performance even under SRS, it is not advisable to use the WaldDiag. The Wald and VCF Wald also exhibited poor performance in the larger models with smaller sample sizes. The Wald test is known to be unstable, mainly when used with data from complex sampling designs and has poor small‐sample behaviour (see e.g. Fay, 1985; Lumley & Scott, 2014). As Fay (1985) and Skinner (2019) have stated, the covariance matrix estimator of the estimated proportions is consistent, but in practice, it will typically be less precise than the multinomial analogues. This reduced precision seriously affects the inversion required in the Wald statistic. In particular, (Fay, 1985, p. 148) wrote that ‘the instability in the estimated inverse, in turn, inflates the rate of rejection under the null hypothesis, often enough to make the test unusable’.

Similar to SRS, the power of the test statistics increased with sample size across all models. Notably, the 2F10V model required a sample size of 5000 to exhibit larger power levels in both sampling designs. The 3F15V model also needed a large sample size under the two‐stage stratified cluster design. For sample sizes of 500 and 1000, the test statistics exhibited low power. It is important to note that in those cases, the Pearson test statistic exhibited power higher than or similar to that of the Wald‐type tests except for the 1F8V model under both designs. The Wald‐type test statistics are possibly affected by the instability of computing and inverting the ${\hat{\Omega }}_{2}$ matrix and the likely sparsity even in the lower order margins in the bigger models and small sample sizes.

**FIGURE A1:**
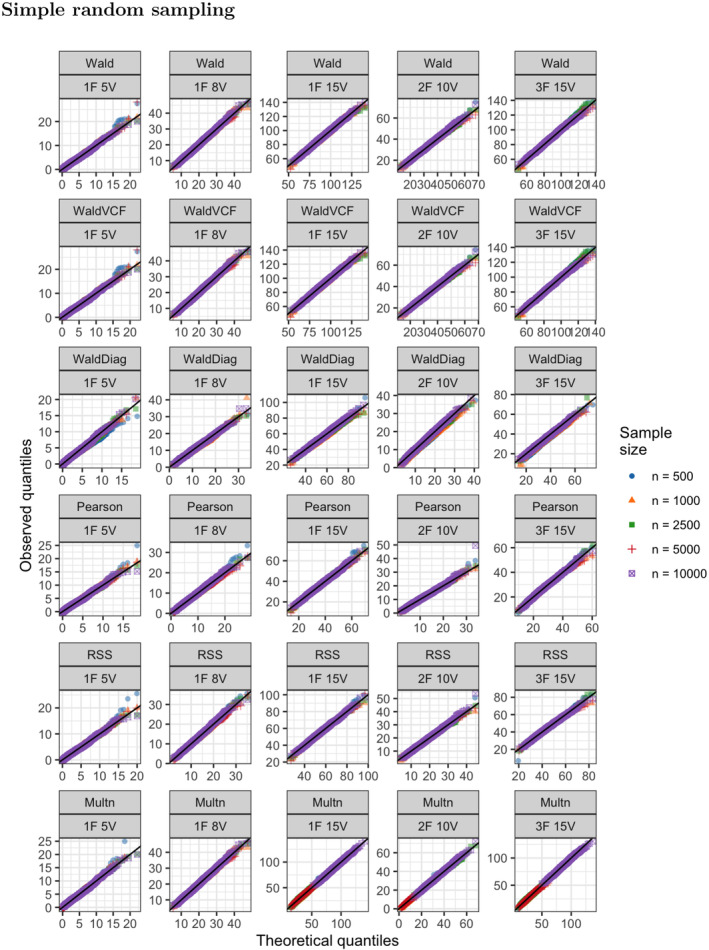
QQ plot for the six test statistics in Table 4 across the five models in Table 3 from simulated data for simple random sampling under the null hypothesis and their respective theoretical asymptotic distributions, n=500,1000,2500,5000, 10,000.

**FIGURE A2:**
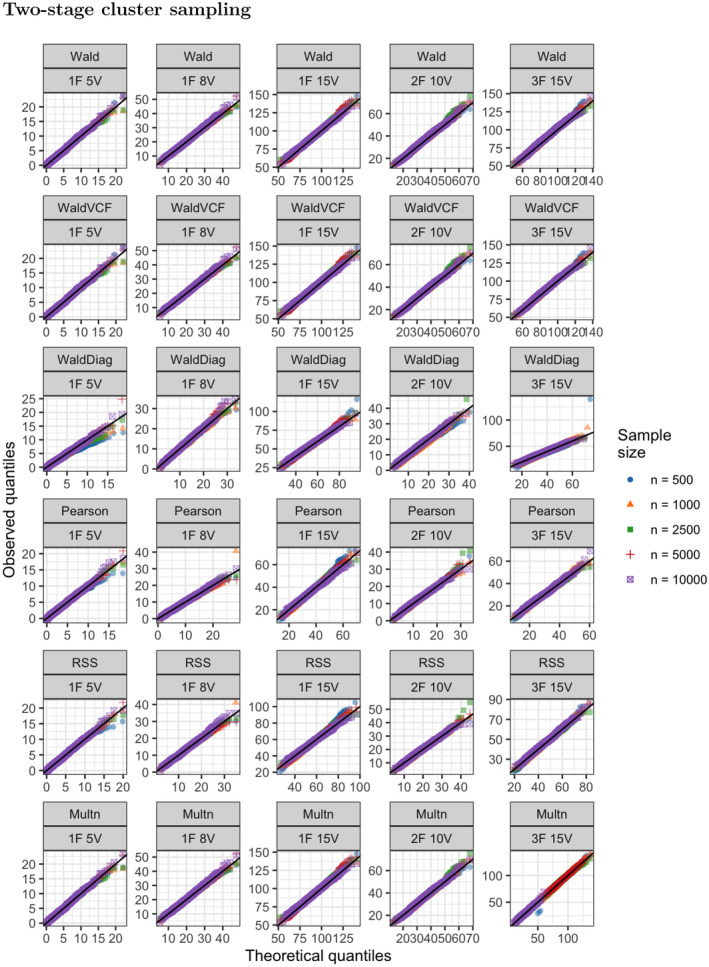
QQ plot for the six test statistics in Table 4 across the five models in Table 3 from simulated data for the two‐stage cluster sampling under the null hypothesis and their respective theoretical asymptotic distributions, n=500,1000,2500,5000, 10,000.

**FIGURE A3:**
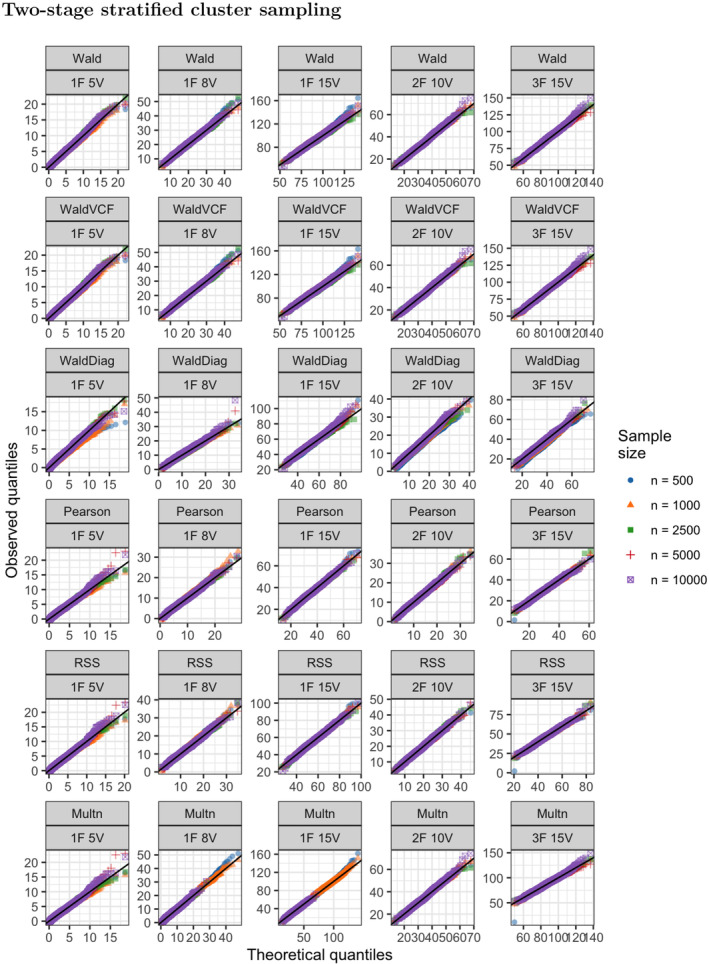
QQ plot for the six test statistics in Table 4 across the five models in Table 3 from simulated data for the two‐stage stratified cluster sampling under the null hypothesis and their respective theoretical asymptotic distributions, n=500,1000,2500,5000, 10,000.

The asymptotic distribution of the proposed test statistics is checked using QQ plots. In Appendix 4, Figures A1, A2, A3 display QQ plots across the 1000 replications for all five models, five sample sizes and simple random sampling, two‐stage cluster sampling, and two‐stage stratified cluster sampling respectively. The plots reveal a generally close alignment between the theoretical distribution of each test and its Monte Carlo distribution. The largest deviations from the asymptotic distribution were found in the WaldDiag test.

## 6 DISCUSSION

This paper expands the pairwise likelihood estimation and limited information test statistics to accommodate complex and informative sampling designs. Specifically, sampling and informative weights are incorporated into the pairwise likelihood to consider the complex design during model estimation and the estimation of asymptotic standard errors. Additionally, the paper introduces limited‐information test statistics of Wald and Pearson's types tailored for complex sampling within the pairwise likelihood estimation framework.

The weighted pairwise likelihood produced unbiased estimates and robustly estimated standard errors. The limited‐information test statistics implemented using pairwise likelihood estimates showed good performance in terms of Type I error and power under simple random and two complex sampling designs, namely, two‐stage cluster and stratified cluster sampling. Although we studied both limited information Wald‐ and Pearson‐type test statistics that work satisfactorily in most cases, the Wald test statistic, for the reason we discussed above, can be unstable, mainly when used to data from complex sampling designs and has poor small‐sample behaviour. Therefore, we recommend using the Pearson test statistic with the pairwise likelihood estimator and the moment matching adjustment. Also, the Pearson test statistic does not require to invert the $\Omega_{2}$ matrix.

One must also consider that sparseness may occur in the lower‐order margins for complex models and small sample sizes. In this case, further investigation is needed to study their performance.

Further research can extend the tests to ordinal responses and investigate resampling methods for estimating the proportions' variance under multistage designs. A Bayesian pairwise approach can also be helpful here for obtaining the test statistics distribution via posterior sampling without relying on the large sample theory presented in this paper.

## AUTHOR CONTRIBUTIONS

**Haziq Jamil:** investigation; writing – review and editing; methodology; software; visualization. **Irini Moustaki:** conceptualization; methodology; validation; formal analysis; writing – original draft. **Chris Skinner:** methodology; writing – review and editing.

## Data Availability

All analyses were conducted in R (R Core Team, 2024) using the accompanying lavaan.bingof (Jamil, 2024) package. The R scripts are available at https://osf.io/2d97y/, including tabulated Type I error rates and power for all simulations conducted.

## APPENDIX 1 PROOF

From (27) we wrote that the score vector can be expressed as a transformation of the $\left(p_{2}-\pi_{2}(\theta )\right)$ vector, which we prove here. Extending (24) to vector form, we have

(A1)
ℝm∋∇ℓP(θ)=n∂πcicj(ij)(θ)∂θki<jk=1,…,m′diagπcicj(ij)(θ)−1pcicj(ij)−πcicj(ij)(θ)i<j=n∂πcicj(ij)(θ)∂θki<jk=1,…,m′diagπcicj(ij)(θ)−1(Bcicj(ij))i<j(p2−π2(θ)),

where ${\left(B_{c_{i}c_{j}}^{\left(ij\right)}\right)}_{i<j}$ is the 4p(p−1)/2×S matrix containing stacked 1×S row vectors $B_{c_{i}c_{j}}^{\left(ij\right)}$ satisfying (26). That is, for any i,j∈{1,…,p} and i<j,

$$\left(\begin{array}{c} B_{00}^{\left(ij\right)}\\ B_{10}^{\left(ij\right)}\\ B_{01}^{\left(ij\right)}\\ B_{11}^{\left(ij\right)} \end{array}\right)(p_{2}-\pi_{2}(\theta ))=\left(\begin{array}{c} p_{00}^{\left(ij\right)}-\pi_{00}^{\left(ij\right)}(\theta )\\ p_{10}^{\left(ij\right)}-\pi_{10}^{\left(ij\right)}(\theta )\\ p_{01}^{\left(ij\right)}-\pi_{01}^{\left(ij\right)}(\theta )\\ p_{11}^{\left(ij\right)}-\pi_{11}^{\left(ij\right)}(\theta ) \end{array}\right).$$

The design should be obvious and simple for the patterns $c_{i}c_{j}=10,01,11$. As for the pattern ‘00’, we may use the fact that the sum of the pairwise proportions is 1:

Combining this design matrix with the preceding terms in (A1), we get

(A2)
B(θ):=∂πcicj(ij)(θ)∂θki<jk=1,…,m′diagπcicj(ij)(θ)−1(Bcicj(ij))i<j,

an m×S matrix dependent on the parameter vector θ. Equation (27) then follows directly.

## APPENDIX 2 ESTIMATION OF DEGREES OF FREEDOM

We discuss the moment matching procedure used to estimate the degrees of freedom of $X^{2}=n{\hat{e}}_{2}^{\prime }\hat{\Xi }{\hat{e}}_{2}$. This statistic has a limiting distribution of $\sum_{s=1}^{S}\delta_{s}U_{s}$, where each $U_{s}\;\overset{\text{iid}}{∼}\;\chi_{1}^{2}$ and $\delta_{s}$ represents the eigenvalues of $M=\Omega_{2}^{1/2}\Xi \Omega_{2}^{1/2}$.

The eigenvalues, which are invariant under cyclic permutations, can be more efficiently computed as the eigenvalues of $\Xi \Omega_{2}$, especially when Ξ is diagonal and $\Omega_{2}$ is dense. In our context, these eigenvalues are estimated using the hat versions of $\Omega_{2}$ and Ξ. For the Wald and VCF Wald test, M becomes idempotent, resulting in an exact chi‐squared distribution for $X^{2}$. Consequently, these estimation procedures are specifically applied to the diagonal Wald and the Pearson test statistics.

Suppose Y follows a $\chi_{c}^{2}$ distribution. We assume $X^{2}$ can be approximately represented as a linear transformation of this chi‐squared random variate:

(A3)
$$X^{2}\approx a+bY.$$

The first three moments of $X^{2}$ are expressed as (Mathai & Provost, 1992, theorem 3.2b.2, p. 53)

(A4)
$$\mu_{1}(X^{2})=\text{tr}(\Xi \Omega_{2}),\mu_{2}(X^{2})=2\text{tr}({\left(\Xi \Omega_{2}\right)}^{2}),\mu_{3}(X^{2})=8\text{tr}({\left(\Xi \Omega_{2}\right)}^{3}).$$

The moments of the approximating random variable a+bY are denoted as

(A5)
$$\mu_{1}(a+bY)=a+bc,\mu_{2}(a+bY)=2b^{2}c,\mu_{3}(a+bY)=8b^{3}c.$$

The formulas for the raw moments of a chi‐squared random variable Y with c degrees of freedom are $\text{E}(Y^{k})=c(c+2)(c+4)\text{⋯}(c+2k-2)$. If we equate these moment expressions and solve for the parameters a, b, and c in the three‐moment adjustment equation, we obtain

$$b=\frac{\mu_{3}(X^{2})}{4\mu_{2}(X^{2})},c=\frac{\mu_{2}(X^{2})}{2b^{2}},a=\text{E}(X^{2})-bc.$$

We can also use two‐moment and one‐moment matching techniques similarly. For the two‐moment adjustment, if we assume $X^{2}\approx bY$, where $Y∼\chi_{c}^{2}$ (i.e. set a=0), the formulae for b and c are as follows:

$$b=\frac{\mu_{2}(X^{2})}{2\mu_{1}(X^{2})}\text{and}c=\frac{\mu_{1}(X^{2})}{b}.$$

On the other hand, in the one‐moment adjustment, let us set c=S, which represents the number of degrees of freedom available for testing. In this case, b can be calculated as $b=\mu_{1}(X^{2})/c$.

In these cases, the tail probabilities can be approximated as

$$\mathrm{Pr}(X^{2}>x)\approx \mathrm{Pr}\left(Y>\left.\frac{x-a}{b}\;\right|\;Y∼\overset{2}{\underset{c}{\chi }}\right).$$

The moment matching procedure is often referred to as the Satterwaithe approximation. The parameter estimates for a,b,c are calculated using estimated versions of Ξ and $\Omega_{2}$ in the given formulas. In this paper, we use the three‐moment procedure due to previous findings suggesting inaccuracies in p‐values obtained from the one‐moment procedure (e.g. Maydeu‐Olivares & Joe, 2008).

## APPENDIX 3 TWO‐STAGE CLUSTER SAMPLING DESIGN, COVERAGE, AND STANDARD ERRORS FOR PARAMETER ESTIMATES

### 3.1

**TABLE A1:** Two‐stage cluster sampling: Coverage rate and the ratio of standard deviation across replications to the estimated asymptotic standard error (SD/SE) for factor loadings and thresholds for the one‐factor model, n=500,1000,5000, weighted and weighted adjusted for clustering pairwise maximum likelihood (PMLW and PMLW‐adj).

	Coverage		SD/SE		Coverage		SD/SE		Coverage		SD/SE	
	n=500				n=1000				n=5000			
	PMLW	PMLW‐adj	PMLW	PMLW‐adj	PMLW	PMLW‐adj	PMLW	PMLW‐adj	PMLW	PMLW‐adj	PMLW	PMLW‐adj
*Loadings*												
$\lambda_{1}$	.94	.92	1.08	1.10	.93	.92	1.09	1.08	.94	.96	1.04	1.00
$\lambda_{2}$	.94	.93	1.07	1.04	.93	.94	1.11	1.05	.93	.95	1.06	.99
$\lambda_{3}$	.93	.93	1.11	1.06	.92	.93	1.14	1.06	.92	.95	1.08	.99
$\lambda_{4}$	.95	.93	1.03	1.03	.94	.94	1.07	1.03	.92	.94	1.08	1.02
$\lambda_{5}$	.92	.90	1.08	1.10	.93	.92	1.08	1.07	.95	.95	1.02	1.00
*Thresholds*												
$\tau_{1}$	.64	.93	2.19	1.08	.67	.95	2.09	1.02	.66	.96	1.99	.97
$\tau_{2}$	.56	.93	2.63	1.05	.57	.95	2.45	.97	.57	.97	2.41	.95
$\tau_{3}$	.70	.94	1.85	1.01	.71	.95	1.86	1.01	.71	.96	1.84	.99
$\tau_{4}$	.80	.94	1.57	1.04	.81	.95	1.45	.96	.80	.96	1.51	.99
$\tau_{5}$	.84	.92	1.38	1.05	.87	.96	1.28	.97	.87	.96	1.31	.98

## References

[bmsp12358-bib-0001] Asparouhov, T. (2005). Sampling weights in latent variable modeling. Structural Equation Modeling, 12(3), 411–434.

[bmsp12358-bib-0002] Bartholomew, D. J. , Knott, M. , & Moustaki, I. (2011). Latent variable models and factor analysis: A unified approach (3rd ed.). John Wiley.

[bmsp12358-bib-0003] Bartholomew, D. J. , & Leung, S. O. (2002). A goodness of fit test for sparse 2^p^ contigency tables. British Journal of Mathematical and Statistical Psychology, 55, 1–15. 12034008 10.1348/000711002159617

[bmsp12358-bib-0004] Bieler, G. S. , & Williams, R. L. (1995). Cluster sampling techniques in quantal response teratology and developmental toxicity studies. Biometrics, 51, 764–776. 7662858

[bmsp12358-bib-0005] Binder, D. A. (1983). On the variances of asymptotically normal estimators from complex surveys. International Statistical Review, 51, 279–292.

[bmsp12358-bib-0006] Cagnone, S. , & Mignani, S. (2007). Assessing the goodness of fit of a latent variable model for binary data. Metron, 65, 337–361.

[bmsp12358-bib-0007] Cai, L. , Maydeu‐Olivares, A. , Coffman, D. L. , & Thissen, D. (2006). Limited information goodness‐of‐fit testing of item response theory models for sparse 2^p^ tables. British Journal of Mathematical and Statistical Psychology, 59, 173–194. 16709285 10.1348/000711005X66419

[bmsp12358-bib-0008] Fay, R. E. (1985). A Jackknifed chi‐squared test for complex samples. Journal of the American Statistical Association, 80, 148–157.

[bmsp12358-bib-0009] Fuller, W. A. (2009). Sampling statistics. John Wiley & Sons.

[bmsp12358-bib-0010] Godambe, V. P. (1960). An optimum property of regular maximum likelihood equation. Annals of Mathematical Statistics, 31, 1208–1211.

[bmsp12358-bib-0011] Hedges, L. V. , & Hedberg, E. C. (2007). Intraclass correlation values for planning group‐randomized trials in education. Educational Evaluation and Policy Analysis, 29(1), 60–87.

[bmsp12358-bib-0012] Jamil, H. (2024). lavaan.bingof: Limited information goodness of fit tests for binary factor models . R package version 0.1.2. https://haziqj.ml/lavaan.bingof/

[bmsp12358-bib-0013] Jöreskog, K. G. (1979). Basic ideas of factor and component analysis. In K. Jöreskog & D. Sörbom (Eds.), Advances in factor analysis and structural equation models (pp. 5–20). Abt Books.

[bmsp12358-bib-0014] Jöreskog, K. G. (1990). New developments in LISREL: Analysis of ordinal variables using polychoric correlations and weighted least squares. Quality and Quantity, 24, 387–404.

[bmsp12358-bib-0015] Jöreskog, K. G. (1994). On the estimation of polychoric correlations and their asymptotic covariance matrix. Psychometrika, 59, 381–389.

[bmsp12358-bib-0016] Jöreskog, K. G. , & Moustaki, I. (2001). Factor analysis of ordinal variables: A comparison of three approaches. Multivariate Behavioral Research, 36, 347–387. 26751181 10.1207/S15327906347-387

[bmsp12358-bib-0017] Katsikatsou, M. (2013). *Composite likelihood estimation for latent variable models with ordinal and continuous or ranking variables* [PhD thesis]. Uppsala University.

[bmsp12358-bib-0018] Katsikatsou, M. , & Moustaki, I. (2016). Pairwise likelihood ratio tests and model selection criteria for structural equation models with ordinal variables. Psychometrika, 81, 1046–1068. 27734296 10.1007/s11336-016-9523-z

[bmsp12358-bib-0019] Katsikatsou, M. , Moustaki, I. , Yang‐Wallentin, F. , & Jöreskog, K. G. (2012). Pairwise likelihood estimation for factor analysis models with ordinal data. Computational Statistics and Data Analysis, 56, 4243–4258.

[bmsp12358-bib-0020] Lindsay, B. (1988). Composite likelihood methods. In N. U. Prabhu (Ed.), Statistical inference from stochastic processes (pp. 221–239). American Mathematical Society.

[bmsp12358-bib-0021] Liu, J. (2007). *Multivariate ordinal data analysis with pairwise likelihood and its extension to SEM* [PhD thesis]. University of California.

[bmsp12358-bib-0022] Lumley, T. , & Scott, A. (2014). Tests for regression models fitted to survey data. Australian & New Zealand Journal of Statistics, 56, 1–14.

[bmsp12358-bib-0023] Mathai, A. M. , & Provost, S. B. (1992). Quadratic forms in random variables: Theory and applications. Dekker.

[bmsp12358-bib-0024] Maydeu‐Olivares, A. , & Joe, H. (2005). Limited and full‐information estimation and goodness‐of‐fit testing in 2^n^ contingency tables: A unified framework. Journal of the American Statistical Association, 6, 1009–1020.

[bmsp12358-bib-0025] Maydeu‐Olivares, A. , & Joe, H. (2006). Limited information goodness‐of‐fit testing in multidimensional contigency tables. Psychometrika, 71, 713–732.

[bmsp12358-bib-0026] Maydeu‐Olivares, A. , & Joe, H. (2008). An overview of limited information goodness‐of‐fit testing in multidimensional contingency tables. In K. Shigemisu, A. Okada, T. Imaizumi, & T. Hoshino (Eds.) New Trends in Psychometrics, (pp. 253–262). Universal Academy Press.

[bmsp12358-bib-0027] Muthén, B. O. (1984). A general structural model with dichotomous, ordered categorical and continuous latent variable indicators. Psychometrika, 49(1), 115–132.

[bmsp12358-bib-0028] Muthén, B. O. , & Satorra, A. (1995). Complex sample data in structural equation modeling. Sociological Methodology, 25, 267–316.

[bmsp12358-bib-0029] R Core Team . (2024). R: A language and environment for statistical computing. R Foundation for Statistical Computing.

[bmsp12358-bib-0030] Reiser, M. (1996). Analysis of residuals for the multinomial item response model. Psychometrika, 61, 509–528.

[bmsp12358-bib-0031] Reiser, M. (2008). Goodness‐of‐fit testing using components based on marginal frequencies of multinomial data. British Journal of Mathematical and Statistical Psychology, 61, 331–360. 17535483 10.1348/000711007X204215

[bmsp12358-bib-0032] Reiser, M. , & VandenBerg, M. (1994). Validity of the chi‐square test in dichotomous variable factor analysis when expected frequencies are small. British Journal of Mathematical and Statistical Psychology, 47, 85–107.

[bmsp12358-bib-0033] Rosseel, Y. (2012). lavaan: An R package for structural equation modeling. Journal of Statistical Software, 48(2), 1–36.

[bmsp12358-bib-0034] Salomaa, H. (1990). Factor analysis of dichotomous data. Statistical studies series #11. Suomen Tilastoseura.

[bmsp12358-bib-0035] Skinner, C. (1989). Domain means, regression and multivariate analysis. In C. Skinner , D. Holt , & T. Smith (Eds.), Analysis of complex surveys. Wiley.

[bmsp12358-bib-0036] Skinner, C. (2019). Analysis of categorical data for complex surveys. International Statistical Review, 87, 64–78.

[bmsp12358-bib-0037] Skrondal, A. , & Rabe‐Hesketh, S. (2004). Generalized latent variable modeling: Multilevel, longitudinal and structural equation models. Chapman & Hall/CRC.

[bmsp12358-bib-0038] Varin, C. (2008). On composite marginal likelihoods. Advances in Statistical Analysis, 92, 1–28.

[bmsp12358-bib-0039] Varin, C. , Reid, N. , & Firth, D. (2011). An overview of composite likelihood methods. Statistica Sinica, 21, 5–42.

[bmsp12358-bib-0040] Williams, R. L. (2000). A note on robust variance estimation for cluster‐correlated data. Biometrics, 56, 645–646. 10877330 10.1111/j.0006-341x.2000.00645.x

[bmsp12358-bib-0041] Wolter, K. (2007). Introduction to variance estimation (2nd ed.). Springer.

[bmsp12358-bib-0042] Xi, N. (2011). *A composite likelihood approach for factor analyzing ordinal data* [PhD thesis]. The Ohio State University.

[bmsp12358-bib-0043] Zhao, Y. , & Joe, H. (2005). Composite likelihood estimation in multivariate data analysis. The Canadian Journal of Statistics, 33(3), 335–356.

